# When “One Size Fits All” Fits None: A Commentary on the Impacts of the“Draft Canadian Breast Cancer Screening Guidelines” on Racialized Populations in Canada

**DOI:** 10.3390/curroncol32030123

**Published:** 2025-02-22

**Authors:** Beverley Osei, Gayathri Naganathan, Juliet M. Daniel, Supriya Kulkarni, Aisha Lofters, Yinka Oladele, Leila Springer, Mojola Omole

**Affiliations:** 1 Temerty Faculty of Medicine, University of Toronto, Toronto, ON M5S 1A1, Canada; beverley.osei@mail.utoronto.ca; 2 Department of Surgery, Michael Garron Hospital, Toronto, ON M4C 3E7 1A1, Canada; gayathri.naganathan@mail.utoronto.ca; 3 Department of Biology, Center for Discovery in Cancer Research, McMaster University, Hamilton, ON L8S 4K1, Canada; danielj@mcmaster.ca; 4 Department of Medical Imaging, University of Toronto, Toronto, ON M5S 1A1, Canada; supriya.kulkarni@uhn.ca; 5 Department of Family and Community Medicine, Women’s College Hospital, University of Toronto, Toronto, ON M5S 1A1, Canada; aisha.lofters@wchospital.ca; 6 African Cancer Support Group, Calgary, AB T1Y 6M6, Canada; info@africancancer.ca; 7 The Olive Branch of Hope, Toronto, ON M3K 1E6, Canada; 8 Department of Surgery, Scarborough Health Network, Scarborough, ON M1P 2T7, Canada

**Keywords:** breast cancer, screening, equity, public health

## Abstract

Epidemiological data show racial and ethnic differences exist in breast cancer morbidity and mortality amongst Black, Indigenous, Asian, and Hispanic populations, with non-white females experiencing earlier age at diagnosis, more aggressive breast cancer subtypes and advanced cancer stages, and earlier mortality than white females. However, the current Canadian breast cancer screening guidelines recommend biannual screening for all females starting from age 50 to age 74 and suggest not to screen individuals aged 40–49. In May 2024, the Canadian Task Force for Preventative Health released updated draft breast cancer screening guidelines, maintaining such recommendations for screening. Both the existing and the proposed guidelines fail to account for the unique cancer burden amongst racialized populations in Canada and risk further perpetuation of existing racial and ethnic disparities by underscreening racialized females. This commentary will present data regarding racial disparities in cancer burden, highlighting the role social and biological factors play in impacting cancer risk and age of disease and presenting perspectives from stakeholder groups reflecting the impacts of current screening guidelines. Ultimately, we critique the current “one-size-fits-all” approach to breast cancer screening in Canada, emphasizing the need for adapted screening practices with the understanding that the current approaches overlook the needs of racialized Canadian populations.

## 1. Introduction

According to Statistics Canada, one in eight females (13%) are expected to be diagnosed with breast cancer in their lifetime [1]. Due to advancements regarding early detection, screening programs, and developments in treatment options, breast cancer mortality rates have declined over the decades, from 41.7 deaths per 100,000 in 1989 to an estimated 21.8 deaths per 100,000 in 2024 [2]. However, despite these developments in cancer research and management, the epidemiological data continue to describe disparities in cancer burden amongst Canadian females, particularly with respect to race and ethnicity, contributing to overt inequities in illness experience and survival outcomes [3].

The literature surrounding the topic of breast cancer largely uses the terminology “women”, a term that refers to an individual’s gender rather than their biological sex. It is important to note that breast cancer can affect populations including cisgender females, transgender men, nonbinary individuals, and other individuals assigned female at birth with no history of bilateral mastectomy, regardless of their gender presentation [2]. Best practices in the literature suggest standardization and precision in the utilization of sex and gender terminology [4,5,6]. Thus, throughout this commentary, we will utilize the term “female” in reference to populations most at risk of developing breast cancer.

The Canadian epidemiological data, along with data from the United States and the United Kingdom, show racial and ethnic differences exist in breast cancer morbidity and mortality amongst Black, Indigenous, Asian, and Hispanic populations [2,7,8,9,10,11,12,13,14,15,16,17]. Although white females have a greater incidence of breast cancer, racialized females are diagnosed at younger ages, present with more aggressive breast cancer subtypes and advanced cancer stages, as well as experience earlier mortality compared with white females [2,7,8,9,10,11,12,13,14,15,16,17].

Despite this, the current Canadian guidelines regarding breast cancer screening for females with average cancer risk recommend biannual (once every 2 years) screening via mammogram for all females starting from age 50 to age 75 and suggest not to screen individuals aged 40–49 [2]. In May 2024, the Canadian Task Force for Preventative Health released updated draft guidelines for breast cancer screening across Canada, reiterating the recommendation for screening to commence at age 50 [2]. Both the existing guidelines and the proposed draft guidelines fail to account for the unique cancer burden amongst racialized populations in Canada and risk the further perpetuation of existing racial and ethnic disparities by underscreening racialized females. We urge the Canadian Task Force to revise their recommendations to account for populations at risk for early-onset and aggressive breast cancer subtypes. This commentary will present the available Canadian data regarding racial disparities in cancer burden and highlight the role that social and biological factors play in impacting cancer risk. We will additionally present the perspectives and recommendations from patient and physician stakeholder groups reflecting the impacts of the current screening guidelines on racialized communities. Ultimately, we will critique the current “one-size-fits-all” approach to breast cancer screening in Canada, supported by existing evidence, with the understanding that such an approach overlooks the unique needs of racialized populations in Canada.

## 2. Current Canadian Screening Guidelines and Rationale

The current Canadian breast cancer screening guidelines recommend that screening commence at age 50 to age 74 for all average-risk females. Additionally, the existing guidelines suggest not to systematically screen with mammography females of ages 40–49. The Canadian Task Force for Preventative Health’s draft update to the breast cancer screening guidelines, released May 2024, reinforces these recommendations. Although the updated draft guidelines note that Canadian data show racial and ethnic variability in incidence, mortality, subtype, and stage at diagnosis within younger age cohorts, they state that there is a lack of data regarding benefits and harms, as well as preferences and values of racialized communities [2]. The task force, therefore, does not make any screening recommendations directed toward racialized groups [2].

Such recommendations do not reflect the current practices in provinces such as PEI [18], Nova Scotia [19], British Columbia [20], and the Yukon, in which mammography is available for individuals starting at age 40. In Alberta [21] and the Northwest Territories [22], breast cancer screening is recommended for individuals from age 45 to 74. As of the Fall of 2024, the Ontario Government has started allowing individuals (females) aged 40 to 49 to self-refer for breast screening mammography through the Ontario Breast Screening Program [23].

In the United States, the U.S. Preventative Services Task Force [24] recently updated their guidelines, reducing the age to initiate biannual screening mammography from age 50 to age 40, and are now consistent with the recommendations of national organizations such as the American College of Radiology [9], the Society of Breast Imaging [25], the American College of Obstetricians and Gynecologists [26], and the National Comprehensive Cancer Network [27]. Similarly, the American Cancer Society [28] recommends breast cancer screening starting at age 45. Such organizations cite growing evidence that screening mammography starting at age 40 can significantly reduce breast cancer deaths [29,30,31]. The American College of Radiology specifically highlights that recommendations to delay screening adversely impact racialized patients as a disproportionate amount of breast cancers are diagnosed before age 50 in Black, Asian, and Hispanic females [32].

Moreover, a U.S. study [33] calculating a risk-adapted breast cancer screening starting age based on 10-year cumulative risk of breast cancer-specific mortality by age, race, and ethnicity identified that while breast cancer screening was recommended at age 50 for the general population, Black females should initiate screening 8 years earlier (age 42), white females at age 51, American Indian or Alaska Native and Hispanic females at age 57 years, and Asian or Pacific Islander females at age 61 years. Additionally, data from the UK Age Trial [34], a randomized controlled trial exploring the impact of mammographic screening before age 50, identified a substantial (order of 25%) reduction in breast cancer mortality with annual screening from age 40. This finding was statistically significant for the first 10 years following randomization and subsequently attenuated (from 20 to 14%). However, the absolute benefit continued to be approximately constant to the end of the follow-up period at 17 years. Their work additionally noted an overdiagnosis rate no greater than that which already occurred from screening at age 50 years and older [34]. Overall, the recommendations and data from national and international organizations, in addition to the clinical practice of various Canadian regions, make clear that screening before age 50 is efficacious, evidence-based, and capable of capturing higher-risk racialized populations.

## 3. Race, Ethnicity, and Ancestry Terminology in Health Demographic Data Collection

We acknowledge that race, ethnicity, and ancestry, although often used interchangeably, are distinct yet interrelated concepts [35]. Race is a social and political concept in which individuals are divided into groups based on perceived physical differences. The use of race in medical practice and health research has falsely substantiated the belief that racial categories reflect biological and genetic differences, which risks the perpetuation of biological determinism and “race science” [36]. However, due to the historic and current impacts of racism, many individuals within a racial category may share common experiences and shared identities. Moreover, race measures can help identify health disparities arising from systemic and structural racism, which is disproportionally faced by populations subject to racialization and racism [36,37].

Ethnicity is a fluid and dynamic term largely used to encompass the complex interrelationship of geography, language, culture, and sense of inclusion [38]. The utilization of ethnicity in health research is contentious, with critics citing its inconsistent and vague use, its potential to oversimplify heterogenous populations, and the risk of contributing to an inappropriate “racialization” of health inequities [39]. Proponents, however, speak to its value when analyzing the relationship between ethnicity and health in the identification of high-risk groups, the development of tailored health services, and the formulation better understandings of the nuances in health behaviours [39]. Ancestry refers to an individual’s or group’s lineage and can refer to geographic origin or genealogic or genetic characteristics and can be linked to the biology of disease development and progression [36]. Due to global migration and displacement patterns, the concept of ancestry can face limitations. As evidenced in this analysis, health disparities are consistently identified along the lines of race, ancestry, and ethnicity, making such terms significant measures to consider in health research.

It is also important to point out flaws inherent in the collection of race-based data, such as the heterogeneity in methods used to collect race, ethnicity, and ancestry data. For example, the cited literature in this paper utilized a variety of methods to categorize race and/or ethnicity, including self-report, country of birth, and inference based on surname. Best practices, as evidenced in the literature, include the use of race and ethnicity data self-reported by the individual; however, even this can be complicated by an individual’s sense of belonging with their heritage community, perceptions of acceptance, and concerns for safety [36]. Moreover, due to an individual’s personal understanding of their racial and ethnic background, as well as constraints present in existing data collection systems, many diverse ethno-cultural populations may be broadly grouped together, limiting nuanced understandings of differences in health behaviours and clinical outcomes. Such considerations further underscore that, however imperfect, race and ethnicity are important and valuable epidemiological measures in health research.

## 4. Racial and Ethnic Disparities in Breast Cancer Burden

Data released by Statistics Canada have outlined details regarding breast cancer incidence, stage, subtype, and mortality in Canada. Such data surrounding breast cancer incidence and stage at diagnosis included data up to 2015, and the mortality findings included data up to 2019. The included racial/ethnic categories were as follows: First Nations, Métis, Inuit, South Asian, Chinese, Black, Filipino, Latin American, Arab, Southeast Asian, West Asian, Korean, Japanese, Multiple Ethnicities, white, and Other. Such ethnic categorizations are utilized by Statistics Canada in its population data collection and, with the exception of “white”, “First Nations”, “Metis”, and “Inuit”, are based on the “visible minority” groups as identified by the Employment Equity Technical Reference Papers, published by Employment and Immigration Canada in 1987 [35]. The term “visible minority”, as utilized by Statistics Canada, is defined by the Employment Equity Act (1995) as “persons, other than Aboriginal peoples, who are non-Caucasian in race or non-white in colour”. According to the Act, Indigenous populations (First Nations, Metis, and Inuit) are considered in the “Not a visible minority” category and are included in a separate category called “Indigenous peoples”. The data were collected using a self-report system, in which individuals were instructed to select one or more of the categories in the provided list and/or instructed to provide a specific response in the “Other” box [35].

The data released by Statistics Canada demonstrate specific racial disparities in breast cancer outcomes. When comparing non-white females with white females, the median age at both diagnosis and death from breast cancer is younger (52 to 60 years compared with 63 years, and 55 years compared with 71 years, respectively) [2]. With respect to stage at diagnosis, Filipina (38.6%), Black (39.2%), South Asian (40.6%), and First Nations (40.7%) females were diagnosed with stage I breast cancer at a significantly lower proportion than white females (46.5%). Additionally, a greater proportion of breast cancers were diagnosed at stage III or IV (26.3%) in Black females than in white females (17%). Among females aged 60–69, both First Nations and Métis females experience a higher mortality rate by 1.13 and 2.58 per 1000 over 10 years, respectively, than white females. Recent data from Wilkinson et al. [7] linking Canadian Cancer Registry and census data similarly identified that non-white females had an earlier peak age of breast cancer diagnosis and a higher proportion of breast cancer cases diagnosed younger than 50 years of age. Moreover, despite a lower incidence of breast cancer, cancer mortality is 40% higher in Black females aged 40–49 years than in white females. Overall, these data indicate that, although the overall lifetime risk of breast cancer is highest in white Canadian females, non-white populations are diagnosed at a younger age, present with more advanced cancer stages, and experience earlier mortality than white females [2,7].

Data suggesting similar disparities have been gathered in the United States and the United Kingdom. In the United States, there is a well-established body of evidence regarding racial inequities in breast cancer outcomes [10,11,12,13]. African American, Native American, Filipino, and Native Hawaiian females living in the U.S. have a greater likelihood of being diagnosed with more advanced malignancies at time of diagnosis and have worse survival than non-Hispanic white females [14,40]. African American females face the greatest mortality rate of any U.S. racial or ethnic group, at a rate 42% higher than that of white American females [11,15,16]. This mortality gap is present at all ages and all stages at diagnosis [41,42].

The relationship between race and age at diagnosis seen in Canadian data is similarly reflected in the U.S. data. The incidence rate of breast cancer presenting before age 45 is greater in Black females than in white females, yet, from age 60 to 84, breast cancer incidence rates are significantly higher in white females than in Black females. Moreover, for patients younger than 50 years, the 10-year survival rate is 64% for Black females which is lower than that for non-Hispanic white Females [43]. In a 2018 study of U.S. cancer registries, Stapleton et al. [32] identified two distinct distribution patterns with respect to age at breast cancer diagnosis, with white patients peaking in their 60s and non-white patients peaking in their 40s.

In the United Kingdom, Caribbean and African females, compared with white females, were identified as significantly more likely to be diagnosed with end-stage breast cancer (OR 1.27 (95% CI 1.12 to 1.43); OR 1.71 (95% CI 1.51 to 1.95), respectively). Asian females were also found to have an increased odds ratio of an end-stage breast cancer diagnosis (OR 1.12 (95% CI 1.03 to 1.22)) [44]. Black females were observed to have worse breast cancer survival than their white English counterparts [45]. A similar trend with respect to cancer subtype is observed, with English females from ethnic minorities (specifically, Indian, Pakistani, Black Caribbean, and Black African) facing greater odds of less favourable breast cancer tumour characteristics, with respect to stage, histological grade, estrogen receptor status, and HER2 status [46].

The reason for such health disparities is multifaceted, involving the complex interplay of individual, disease, social, and systemic factors. Ultimately, these data suggest that practices initiating breast cancer screening at age 50 risk disadvantaging populations with greater proportions of breast cancer presenting before the age of 50 and may partially explain the racial and ethnic disparities in advanced-stage breast cancer at diagnosis identified in the current data [7].

## 5. Biology, Ancestry, and Breast Cancer

With respect to the biological factors impacting breast cancer burden, there is evidence that the incidence of triple-negative breast cancer (TNBC), a tumour subtype associated with poor outcomes and reduced survival, is much greater in Black [47,48,49,50,51,52,53] and Hispanic [54] females than in white females. TNBC is a breast tumour subtype that is negative for estrogen receptor (ER), progesterone receptor (PR), and human epidermal growth factor receptor-2 (HER2) expression [54,55]. It is associated with high proliferative indices, propensity for brain metastasis, high recurrence rates, and a lack of response to hormonal (Tamoxifen) or anti-Her2 therapies [55]. TNBC additionally presents at disproportionately high rates among younger individuals, with data showing that patients less than 40 years of age were 1.5 times more likely than 60- to 69-year-old patients to be diagnosed with TNBC [54]. Population-based data from the United States have indicated a two-fold greater incidence rate of TNBC amongst African American females than in white females across all age cohorts [48,56].

In a systematic review and metanalysis of studies on breast cancer amongst African females, Hercules et al. identified that TNBC frequency was greatest in West African populations (45.7%), compared with other regions in the African continent [57]. Stark et al. [52] reported a TNBC prevalence of 82% amongst breast cancer samples of Ghanaian patients, compared with the U.S., in which the prevalence rates were 33% for African American and 10% for white American females. In a study by Agboola et al. [58], in which breast cancer samples, clinical histories, and tumour characteristics were compared between Nigerian females and a matched white UK-based cohort, a greater incidence of TNBC was identified in Black females from Nigeria (approximately 48%), than in white females from the UK [58]. Moreover, patients from the Nigerian cohort were found to present more frequently with premenopausal status, large primary tumour size, high tumour grade, advanced lymph node stage, and a higher rate of vascular invasion [58]. The frequency of TNBC prevalence appears consistent with rates observed in other populations with significant West African ancestry, such as within the Caribbean [59,60], the United Kingdom [61], and North America [62,63].

Furthermore, the transcription factor Kaiso (ZBTB33) has been hypothesized to play a role in impacting mortality outcomes in people of African ancestry with cancer, due to data displaying associations between its increased expression and poor overall survival in African American individuals with breast and prostate cancer [64,65]. It has additionally been purported to serve a significant role in the development of aggressive cancer subtypes, such as TNBC [66,67]. Studies performed by Bassey-Archibong et al. [68], as well as Jones et al. [64], reported an overexpression of nuclear Kaiso in breast tumour tissue samples of patients with West African ancestry (Nigerian, Barbadian, and African American), compared with that in white patients. Although there is as yet no literature describing population-level data, it remains an important genetic contributor to consider for breast cancer risk amongst females with West African ancestry. The increased expression of Kaiso, along with the increased prevalence of TNBC in both West African and West African diasporic patients, suggests that West African ancestry may be associated with a genetic predisposition for early-onset, high-risk breast malignancy [52,68]. Given the prevalence of individuals of West African descent in Canada, it is vital that any implemented breast cancer screening interventions remain acutely aware of the identified associations of this ancestry with high-risk breast cancer subtypes and presentations.

## 6. Stakeholder Perspectives and Recommendations

The need for updated breast cancer screening guidelines that account for the needs of high-risk racialized populations is further reinforced by stakeholder perspectives. Such groups have released statements in response to the Canadian Task Force’s draft recommendations. These responses have by and large critiqued the apparent disregard towards the benefits of a lowered screening age in strengthening earlier detection, diagnosis, and overall outcomes. Such groups include Canadian organizations such as the Canadian Society for Breast Imaging [69], the Canadian Cancer Society [70], and the Canadian Association of Radiologists [71]. The Canadian Society of Breast Imaging particularly notes in their statement that breast screening programs that begin at age 50 delay diagnosis in racialized females [69]. Organizations such as Breast Cancer Canada, a national organization with a focus on advancing breast cancer research, showed concerns regarding the new draft guidelines, urging in their statement that the task force reconsider their recommended guidelines [72]. In their statement, the charity notes survey data conducted by the organization revealing significant national demand for earlier breast cancer screening, with 89% of surveyed Canadians believing that routine screening should be initiated before age 50. Breast Cancer Canada additionally highlights the urgent need for more robust race-based data collection in Canada, with 79% of surveyed Canadians believing Canada should make gathering race-based data on cancer screening rates a priority, addressing the current dearth of information regarding disparities in breast cancer care [73]. Patient advocacy groups, such as the Canadian Cancer Survivor Network, have expressed “disappoint[ment] and outrag[e]” towards the task force’s recommendations, noting that the current screening guidelines contribute to a significant portion of the population being missed [74]. Overall, we agree with the stakeholders’ expressed concern and urgency for screening services that recognize and account for those most in need of targeted care within our healthcare systems.

## 7. Recommendations

Our recommendations are as follows:

(1) Revision of the Canadian Task Force screening recommendations to begin at breast cancer screening at age 40, consistent with other OECD jurisdictions. Screening this age group (ages 40–49) will reflect age-specific patterns based on race/ethnicity and account for groups at higher risk of early-onset breast malignancies.
(2) Development of risk assessment guidelines that consider West African ancestry as a risk factor for more aggressive cancer subtypes, such as TNBC, to guide personalized screening strategies. Such a recommendation aligns with the recommendations of the American College of Radiologists, who suggest all females over the age of 25 should undergo risk assessment to allow for appropriate planning for screening onset, particularly for Black females and those of Ashkenazi Jewish descent, who are at higher risk for genetic mutations, and for Black and other racialized females who are at higher risk for early-onset breast cancers [14].
(3) Investment in population-level data collection and analysis regarding racial disparities in cancer screening and the impact of systematic early screening programs on racialized populations

Canadian data, along with the literature from the U.S. and the UK, have established that racialized females are more likely to be diagnosed with breast cancer at a younger age, present with more aggressive cancer subtypes, present at more advanced disease stage, and experience higher mortality [2,8,10,11,12,14,16,32,41,44,75]. Females of West African descent in particular have a greater likelihood of early presentation and diagnosis of triple negative breast cancer than other ethnic groups. Such disparities make clear that a “one-size-fits-all” approach to breast cancer screening is limited and, thus, inappropriate in accounting for the needs of racialized populations in Canada.

The findings gathered in the literature, along with the advocacy of physician and patient groups, have challenged established practices with respect to breast cancer screening and provide evidence that specific race/ethnicity-based considerations need to be made for screening and risk stratification guidelines. As early detection of cancer is vital to treatment effectiveness and improved survival [76], considerations must be made for the populations that present with breast cancer at a younger age.

Although further race- and ethnicity-based data are needed in Canada to explore the specific biological and social determinants that influence disparities in cancer burden and outcomes, the wait for such data and their analysis cannot be used to fuel further inaction. This is particularly the case when such waiting comes at the cost of the improved health and quality of life of racialized populations in Canada.

## Abbreviation

The following abbreviations are used in this manuscript:

TNBC	Triple-negative breast cancer
